# European Echinococcosis Registry: Human Alveolar Echinococcosis, Europe, 1982–2000

**DOI:** 10.3201/eid0903.020341

**Published:** 2003-03

**Authors:** Petra Kern, Karine Bardonnet, Elisabeth Renner, Herbert Auer, Zbigniew Pawlowski, Rudolf W. Ammann, Dominique A. Vuitton, Peter Kern, the European Echinococcosis Registry

**Affiliations:** * University of Ulm, Ulm, Germany; † University Hospital, Besançon, France; ‡ University Hospital Zürich, Zürich, Switzerland; § University of Vienna, Vienna, Austria; ¶ A. Wrzosek Collegium, Poznan, Poland; # University of Franche Comté, Besançon, France; ** University Hospital Ulm, Ulm, Germany

**Keywords:** alveolar echinococcosis, alveolar hydatid disease, Echinococcus multilocularis, epidemiology, geographic distribution, risk factors, clinical characteristics, case registry, research

## Abstract

Surveillance for alveolar echinococcosis in central Europe was initiated in 1998. On a voluntary basis, 559 patients were reported to the registry. Most cases originated from rural communities in regions from eastern France to western Austria; single cases were reported far away from the disease-“endemic” zone throughout central Europe. Of 210 patients, 61.4% were involved in vocational or part-time farming, gardening, forestry, or hunting. Patients were diagnosed at a mean age of 52.5 years; 78% had symptoms. Alveolar echinococcosis primarily manifested as a liver disease. Of the 559 patients, 190 (34%) were already affected by spread of the parasitic larval tissue. Of 408 (73%) patients alive in 2000, 4.9% were cured. The increasing prevalence of *Echinococcus multilocularis* in foxes in rural and urban areas of central Europe and the occurrence of cases outside the alveolar echinococcosis–endemic regions suggest that this disease deserves increased attention.

Human alveolar echinococcosis, caused by the metacestode of the fox tapeworm *Echinococcus multilocularis*, is considered to be the most pathogenic zoonosis in temperate and arctic regions of the Northern Hemisphere. Transmission to humans occurs when eggs of the tapeworm, excreted by the final hosts (usually foxes), are accidentally ingested. The larva’s primary target organ is the liver, where it proliferates slowly, but the larva also spreads into extrahepatic structures and even metastasizes to distant organs. In earlier cohorts, the fatality rate exceeded 90% within 10 years (*1*). The introduction of benzimidazoles for alveolar echinococcosis treatment in 1976 has considerably improved the prognosis (*2*,*3*). Long-term follow-up of 117 patients showed that the 5-year actuarial survival rate increased to 88% with this improved management (*4*). As chemotherapy is parasitostatic only, long-term administration is mandatory for most patients (*5*,*6*). Radical surgical excision, the only curative treatment, is feasible in a few select cases (*7*).

In Europe, previous assessments of human cases did not cover all alveolar echinococcosis–endemic areas at comparable periods. In Switzerland, where laboratory-diagnosed alveolar echinococcosis was a reportable disease until 1997, the annual incidence ranged from 7.2 to 10.4 (0.10–0.18/100,000) and did not markedly vary during a 36-year period (*8*). In Austria, an average incidence of 2.5 cases per year corresponded to an incidence of 0.034/100,000 from 1985 to 1999 (*9*). These low numbers of human infections throughout a whole country failed to alarm public health authorities. However, two findings are beginning to attract more attention: 1) high annual incidence rates occurring regularly in particular regions, e.g., the Swiss Jura (0.74/100,000) (*10*); and 2) a presumed range extension of the parasite in its sylvatic life cycle.

In Europe, the Red Fox (*Vulpes vulpes*) is the most important final host for *E. multilocularis*. Reviews based on the data collected during the past decade have shown that the natural range of the parasite extends farther to the east and north in Europe than previously thought (*11*,*12*). Defined rural areas have been monitored regularly for many years, and increasing parasite prevalence rates in foxes have been recorded (*13*). Clusters of high endemicity (60% to 75%) have been found (*14*). Increasing fox populations have been reported from several European countries (*13*). Foxes migrating to urban areas are also causing concerns: *E. multilocularis* prevalence rates of 20% and 48% have been recorded in Stuttgart, Germany (*11*), and Zürich, Switzerland, respectively (*15*).

Knowledge of the parasite’s range and prevalence in animal hosts has thus grown during recent years. However, comprehensive assessments of human alveolar echinococcosis covering the known risk areas across European countries have not been performed. To provide baseline data for future risk calculations and to establish a prospective case retrieval, the European Echinococcosis Registry (EurEchinoReg) created a network of reporting centers in 11 countries of western and central Europe and Turkey. This report provides the status of reporting, origin, and clinical and epidemiologic data of such patients reported to the registry up to the year 2000.

## Methods

### Case Retrieval

Case detection and data collection have been organized by each participating country according to the existing infrastructure of the national health systems and the availability of data sources. In the EurEchinoReg, experts from universities (research units and hospitals) and public health authorities cooperate in eight countries of the European Union (Austria, Belgium, France, Germany, Greece, Great Britain, Italy, and the Netherlands), and in Switzerland, Poland, the Czech Republic, and Turkey. Patient data are stripped of identifiers and sent to two subregistries (the University of Franche-Comté, Besançon, France and the University of Ulm, Ulm, Germany), where they are controlled and approved for electronic recording.

### Case Definition and Period of Inclusion

Diagnosis of alveolar echinococcosis is confirmed by 1) positive histopathology, if available and/or 2) typical liver lesion morphology identified by imaging techniques (ultrasound scan, computed tomography, and magnetic resonance imaging) with or without the detection of serum antibodies (serology). Positive serologic results without suggestive imaging findings or positive histopathology does not qualify for a case definition.

The period of inclusion began in 1982, when benzimidazoles, ultrasound, and other imaging techniques (which facilitated diagnosis, treatment, and follow-up) were introduced. The registry includes all confirmed new cases from January 1982 to December 2000, as well as cases with a diagnosis from earlier periods, provided the patients were alive in 1982 and their diagnosis was confirmed with the appropriate techniques in 1982 or later.

### Case Report Form and Completeness of Registration

Patients were asked to allow their nominal registration at their national center, in conformity with the national legislation for data privacy to avoid double registration and to facilitate follow-up. Two questionnaires are used: an epidemiologic part to be answered by the patient and a clinical part to be completed by the reporting physician. In addition to demographic baseline data, we gathered information on year of diagnosis, disease manifestation at the time of the diagnosis, co-existing conditions; diagnostic and therapeutic measures, year of death, presumed cause of death, places of residence, and occupation in agriculture, forestry, and gardening.

Data files from the study groups in Austria, France, Germany, and Switzerland were the basis for the European patient registry; additional case files were collected by active case finding and with the help of physicians from hospitals and private practices. Completeness of registration can be assumed: 1) in France, since access to patient files is facilitated by a centralized distribution of albendazole by a few university hospitals; 2) in Austria, since laboratory diagnosis is made in a single institution; and 3) in Switzerland, where alveolar echinococcosis was a reportable disease until 1997; case reports are thus complete from the 1970s until 1997. Underreporting is likely in Germany, where reporting relies entirely on the cooperation of family physicians and clinicians. In Belgium, Greece, and Poland, alveolar echinococcosis seems to be newly emerging, and cases are discussed in the medical community; the cases reported to the registry should reflect the true prevalence in these countries.

### Data Analysis

The combined data sets for all European patients are kept in an Access database (Microsoft Corp., Redmond, WA). Descriptive analyses were made with SAS software V8 (SAS Institute, Inc., Cary, NC). The regional distribution of alveolar echinococcosis cases was mapped with the software package RegioGraph 5.1 (GfK MACON AG, Waghãusel, Germany).

## Results

### Epidemiology

The total number of verified alveolar echinococcosis cases reported to the registry was 559; 42.0% were diagnosed in France, 23.6% in Germany, and 21.1% in Switzerland (Table 1). Fifteen patients acquired the infection outside their reporting country, 7 of these cases originated from one of the neighboring countries, 8 were of non-European origin.

**Table 1:** Number of patients with alveolar echinococcosis, Europe

Yr of first diagnosis	Reporting country									
	Austria	Belgium	France	Germany	Great Britain	Netherlands	Switzerland	Poland	Greece	Total^a^
Until 1980	8	0	23	30	0	0	43	0	0	104
1981–1985	12	0	60	11	0	0	29	0	0	112
1986–1990	11	0	80	17	0	0	17	2	0	127
1991–1995	13	0	40	26	0	0	16	6	0	101
1996–2000	10	3	32	48	1	1	13	6	1	115
Total no. of patients^b^	54 (1)	3	235	132(6)	1 (1)	1 (1)	118 (6)	14	1	559

^a^Status of notification to the European Echinococcosis Registry as of August 2001. ^b^Includes 15 non-autochthonous cases (in parentheses); 7 of them originated from neighboring countries, 3 from Turkey, 3 from the Newly Independent States, 1 from Kazakhstan, and 1 from Afghanistan.

**Figure 1:**
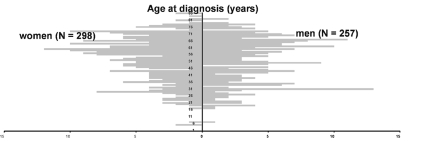
Patients with alveolar echinococcosis reported to the European Registry. Age at first diagnosis by gender (N=555, year of birth missing for 4 patients).

During the reporting period, the number of new cases varied from year to year. From 1981 to 2000, a peak incidence of 36 was noted in 1988; aside from this 1 year, reports ranged from 15 to 27 patients. A total of 258 patients were male (46.2%) and 301 female (53.8%) (gender ratio 1:1.2). The median age at first diagnosis was 56 (mean 52.5, range 5–86 years) and was almost equal in men and women (Figure 1). The proportion of patients <20 years old was 2.1% (12/559); 88 (15.7%) were >69 years of age. For four patients (0.7%), the year of birth was missing. Three of the four children in this case series, ages 5 and 7 when diagnosed, had severe organ damage; two were immunocompromised.

Information on potential risk factors was available for 210 (37.6%) patients from Austria, Germany, Greece, and France (Table 2), including 97 men and 113 women. Of these, 21.9% were farmers. In addition, of all the patients engaged in other professions (including housewives and students), 46.2% regularly farmed, gardened, or performed related activities as a pastime. Of all pensioners and unemployed patients, 62.2% also gardened, farmed, or the like. Most patients (70.5%) owned or formerly kept dogs and cats. Among these pet owners, 105 persons also actively farmed or gardened. Only 15 patients (7.1%) did not farm, garden, or own pets.

**Table 2:** Possible exposure risks assessed for 210 patients with alveolar echinococcosis

Occupation	N (%)	Activity in agriculture, gardening, forestry, hunting			Ownership of dogs, cats, or both			
		Yes	No	Missing	Yes	No		Missing
Farmers	46 (21.9)	46	0	0	39	2		5
Nonfarmers^a^	119 (56.7)	55	56	8	80	13		26
Occupation not specified, including unemployed and pensioners	45 (21.4)	28	13	4	29	6		10
Total	210 (100.0)	129	69	12	148	21		41

^a^For example, tailors, hairdressers, cooks, nurses, drivers, teachers, students, and housewives.

### Geographic Distribution

**Figure 2:**
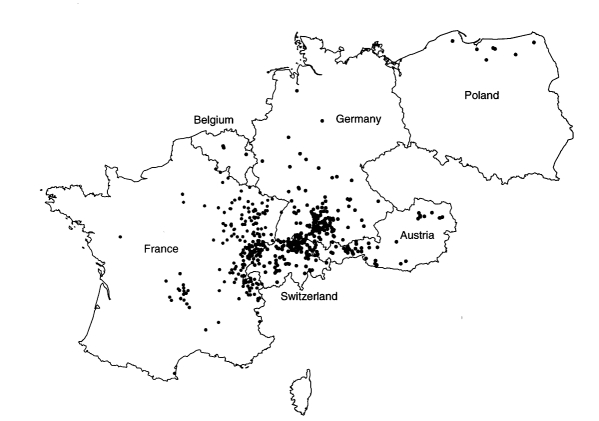
Regional distribution of autochthonous alveolar echinococcosis in Europe, from 532 diagnoses ascertained from 1982 to 2000. Dots represent place of residence (at time of diagnosis or last medical record) of 1–5 patients. In Austria, Belgium, Germany, and Poland, administrative units for locating patients are the municipality; in France and Switzerland, dots are placed at random in larger units (“Arrondissement” for France, “Kanton” for Switzerland). Source: European Echinococcosis Registry, Ulm, Besançon, 2001. Used with permission.

Figure 2 gives the residence at the time of diagnosis or at the time of the last medical report for 532 alveolar echinococcosis patients; cases were autochthonous from the countries represented on the map. The patient from Greece lived in Macedonia. Data were unavailable for 18 patients.

Most residences were clustered in defined regions: central France, French Jura and Savoy, Swiss Jura and northeastern Switzerland, southern Germany, and western Austria. Single cases were identified in Belgium, the northern regions of France, Germany, and Poland, and northeastern Austria. For the period 1980–1999, a total of 201 cases were reported from Turkey; all originated from the Asian part of the country, mostly from eastern Anatolia. However, the aggregated data (reviewed by Altintas et al. [*16*]) could not be combined with the detailed datasets from western and central Europe. No autochthonous cases were reported from the Netherlands, the Czech Republic, the Slovak Republic, Italy, or the U.K.

### Clinical Data

The main diagnostic procedures, conducted within a time span of 6 months after initial examination, which led to the diagnosis of alveolar echinococcosis (Table 3). A total of 53.5% of diagnoses were definitely confirmed by positive histopathology; 38.5% were ascertained by imaging techniques combined with serology, or imaging alone, when obtaining tissue specimens for analysis was not possible. Information on diagnostic procedures was missing for 7.7% of the patients.

**Table 3:** Diagnostic procedures to ascertain the diagnosis of alveolar echinococcosis^a^

Histopathology^b^		Imaging^c^		Serologyd	No. of patients (%)
+		+		+	176 (31.5)
+		+		–	48 (8.6)
+		–		+	19 (3.4)
+		–		–	56 (10.0)
Subtotal					299 (53.5)
-	+		+		192 (34.3)
-	+		-		25 (4.5)
Subtotal					217 (38.8)
Data not available					43 (7.7)
Total					559 (100.0)

aAll documented techniques, applied during 6 months after initial examination. +, positive result in the respective tests/examinations; –, negative result, tests/examinations not done, or data not available. ^b^Examination of liver tissue samples carried out on material removed by surgery, diagnostic laparoscopy, or, in rare instances, by fine needle biopsy. ^c^Comprised one or several examinations, i.e., ultrasound, computed tomography (CT), or magnetic resonance imagry (MRI) of the abdomen. In some cases, X-ray, CT, or MRI were available on brain, chest, or other organs. dIncluded screening methods using different crude antigen preparations in indirect hemagglutination or enzyme-linked immunosorbent assays (ELISA). In addition, purified and recombinant antigen preparations such as Em2+, Em10 or Em18 were used in ELISA, Western blot, or both.

In 397 (71.0%) of the 559 cases, the diagnosis was made after the patients reported symptoms; 66 (11.8%) cases were disclosed by chance in the course of a general medical check-up or an examination related to other diseases; and 18 (3.2%) cases were found during studies that screened for alveolar echinococcosis. Data on these circumstances were not available for 78 (14%) of 559 patients.

The primary infection site was the liver for almost all patients, and primary extrahepatic lesions without any involvement of the liver were diagnosed in 13 patients (Table 4). At first diagnosis, the liver was the only affected organ for 351 (62.8%) of 559 patients. Damage to the liver included single or multiple lesions in one or more segments of one or both liver lobes, the hilus region, the intrahepatic portal vein, hepatic vein, or bile duct. Approximately one third (34%) of the patients (190/559) were already affected by a spread of the larval tissue either in continuum into neighboring organs, by the formation of distant metastases, or both. Specific details of organ damage were available for 178 of 190 patients. The organs most frequently affected by continuous growth were the diaphragm (59 patients), kidneys or adrenal glands (26 patients), and lungs and pleura (15 patients). Metastases occurred mainly in the lungs (39 patients), brain (17 patients), and spleen (10 patients).

**Table 4:** Location of the primary lesions at first diagnosis in alveolar echinococcosis

Primary infection site	No. of patients
Liver	541 (96.8%)
Spleen, peritoneum, lung, vertebra, brain, kidneys, heart	13 (2.3%)
Data not available	5 (0.9%)
Total	559

At the time of reporting, 267 (47.8%) of the 559 patients had undergone surgery and received benzimidazoles; 200 (35.8%) were treated with these drugs alone, and 48 (8.6%) by surgery alone. A total of 29 patients (5.2%) underwent liver transplantation. Twenty-two patients (3.9%) had not received any treatment during the time between diagnosis and notification; another 13 (2.3%) had apparently had inadequate treatment. For 9 patients (1.6%) the chosen treatment options were not specified.

By December 2000, 73.0% of the patients were alive, 21.3% had died, and 5.7% were lost to follow-up (Table 5). Of the patients still alive, disease activity was assessed at their last clinical examination as follows: cured (20 patients, 4.9%), stable or regressive (226 patients, 55.4%). Progression, sequelae, or complications caused by larval growth or occurring after intervention were indicated in 43 cases (10.5%). An assessment was not provided for 119 patients (29.2%); many of them had been diagnosed recently, and treatment had just begun. Death was definitely associated with alveolar echinococcosis in 13 (10.9%) of the 119 cases; in 15 cases (12.6%) death was probably related to this disease. In 20 patients (16.8%), death was definitely independent of the diagnosis of alveolar echinococcosis. No assessment was available for 71 patients (59.7%).

**Table 5:** Vital status of patients with alveolar echinococcosis, as of December 2000

Yr of first diagnosis	No. of patients			Interval between diagnosis and death (yrs)
	Alive	Lost to follow-up	Deceased	
Until 1980	63	4	37	4–29
1981–1985	73	11	28	<1–14
1986–1990	84	9	34	<1–10
1991–1995	85	6	10	1–4
1996–2000	103	2	10	<1–1
Total	408	32	119	

## Discussion

In 1998, the EurEchinoReg network initiated the assessment of human alveolar echinococcosis across European borders. The reasons for promoting concerted efforts to survey a disease thought to be rare in Europe were as follows: 1) the disease is one of the most aggressive chronic liver diseases, 2) comprehensive assessments of human cases covering all known risk areas were not available, 3) the routes of transmission to humans are still hypothetical, and 4) the range of the parasite in its life cycle seems to have extended, posing threats in previously unaffected areas.

The assessment included patients from former clinical studies and cases identified by active case finding. Nine European countries reported on 559 patients; cases were autochthonous from seven of these countries. The median numbers per year did not vary during two decades (24 in 1980s; 22 in 1990s). Underreporting from previous years was responsible for a seemingly increasing incidence in Germany; underreporting since 1998 explains a decline in Switzerland. High numbers in France in the mid-1980s could be an effect of mass screenings performed at that time in alveolar echinococcosis–endemic areas, which may have raised awareness of the disease. In the past, the number of verified and published cases from Europe (Austria, France, Germany, and Switzerland) amounted to 844 cases or 10.6 cases per year (published between 1900 and 1980) (*17*). The patient numbers from our report reflect what is probably an optimal detection rate owing to improved technology. Thus, low but constant incidence is characteristic of the occurrence of human alveolar echinococcosis in Europe today.

In this parasitic infection, a long incubation period seems to precede diagnosis. Albeit difficult to prove, the initial asymptomatic period is assumed to last 5–15 years (*1*). (This conclusion is derived from the small proportion of patients <20 years old at diagnosis [2.1% in this report]). Determining the time and place of infection is difficult. Assuming that in humans, who are unsuitable hosts for *E. multilocularis*, repeated or long-term exposure is required before an infection becomes established, these conditions are more likely to be met by outdoor activities close to the place of residence than by travels to alveolar echinococcosis–endemic areas. We therefore assume that for most cases, the place of residence is most likely the area of infection. A complete documentation of all the places where the patients had lived during their lives was available for approximately 30%. Mobility of this patient subgroup was low, in conjunction with long-term farming.

The distribution of alveolar echinococcosis in Europe shows a core area with a high density of cases and border areas with clusters of a few patients or single cases. The core area covers large parts of the classic alveolar echinococcosis–endemic regions in Austria, France, Germany, and Switzerland, including those where the index cases from each of these countries have been identified since 1855 (*17*). In these areas, recent screening studies have detected not only a small number of manifest diseases but also self-cured infections (aborted lesions, first described by Rausch et al. [*18*]), and seropositivity rates of up to 2% (*14*,*19*,*20*). Fifteen persons with aborted hepatic lesions (lesions with characteristic calcification) and positive serologic results were reported to the registry but were excluded from this analysis, since a definite diagnosis based on histopathologic or molecular findings had not been provided. Together with a persistent *E. multilocularis* seroprevalence, such reports point to a manifest infection pressure in the core area.

In the core area, a consistently high prevalence of *E. multilocularis* in foxes has been reported, e.g., >50% in southwestern Germany (*11*), 44% in western Bavaria (*21*), 65% in eastern France (*13*), and 35% in western Austria (*9*). In the border area with less frequent and more dispersed human cases, fewer investigations have been undertaken to establish parasite prevalence, and the figures determined rely on low numbers of examined foxes. The prevalence was generally low, e.g., 13% in northern Germany (*11*), 8% to 21% in eastern Bavaria (*21*), and 10% in eastern Austria (*9*). In Belgium, the first three patients with autochthonous infections lived in areas with low parasite prevalence (final report to the European Commission, Directorate General V (EurEchinoReg, unpub. data, 1999). No prevalence data are available for northern France. In Poland, parasite prevalence was initially investigated in 1994. All registered patients live in the northeastern districts with the highest prevalence (20% and 36%) (*22*). In Greece, sporadic cases had been reported previously (*23*).

Whether low parasite prevalence exerts an infection pressure relevant for transmission to humans remains questionable. Recent investigations have shown that foci of high prevalence can persist, even for long periods, in regions where the overall infection rates in foxes are low (e.g., foci of 25% in areas with 5% in eastern Germany) (*24*). Similar foci may exist in other regions but are undetected to date. Thus, human infection can probably occur in regions with low overall parasite prevalence, and we regard case reports from areas remote from the core area as strong hints of new areas at risk. Therefore, threshold findings at diagnosis should not be rated as incompatible with the disease when the patient lived in an area where human alveolar echinococcosis was unknown before. According to Eckert et al. (*12*), all regions with a proven occurrence of *E. multilocularis* in Red Foxes indicate a “potential risk area,” irrespective of the magnitude of prevalence rates. This view is the basis for the current concept of a continuous distribution of the parasite in Europe from central France to Poland. Future studies should, therefore, address the redefinition of risk areas for alveolar echinococcosis and the population at risk.

Transmission of the parasite to humans occurs rarely, and individual risk factors for human disease are not well understood. In Europe, only one case-control study has been published; this study included 21 patients and 84 controls from Austria (*25*). A high association of the disease was found with cat ownership and hunting, but because of the low case number the study was of limited power. Farming did not seem to have an impact on infection risk. In China, a population-based study showed that farming was the most important risk factor (*26*). In Alaska, dog ownership was found to be associated with the disease (19 patients, 38 controls) (*27*). None of these studies found an association of the disease with a history of picking and eating wild berries and mushrooms or raw produce from unfenced gardens. Also, neither fox hunters in China nor trappers in South Dakota, United States, are affected by the disease (*28*). The records of 210 patients from the European registry data show that 21.9% were farmers; another 39.5% were engaged in farming, gardening, hunting, or working in forestry as a pastime; 70.5% of all patients kept dogs or cats. These data point to a high frequency of putative exposure, but the lack of a comparison group does not allow an evaluation of the risk potential of these activities. These activities may be characteristic of most people in rural communities in Europe. For Europe, the questions of how risk behavior can be defined and how exposure can best be prevented are, therefore, still unanswered.

Within the last 20 years, major improvements have been made in the diagnosis and treatment of alveolar echinococcosis. Definite diagnosis by histopathology was available in 53.3% of this case series; the remaining cases were ascertained by imaging with or without specific serology. At diagnosis, 34% of the patients were already affected by advanced larval growth; when the parasitic tissue does not affect important organs or vessels, it may go unnoticed for prolonged periods. This fact may also explain a diagnosis late in a patient’s life. In Europe, the mean age at diagnosis was slightly higher than in non-Caucasian populations, i.e., in Hokkaido, Japan (48.7 years) (*29*), or in China (35.7 years) (*30*).

Immunodeficiencies, e.g. HIV infection (*31*) or immunosuppressive therapy after liver transplantation (*32*), may possibly accelerate the manifestation of alveolar echinococcosis. Chemotherapy with benzimidazoles was the only treatment for one third of the registered patients; complete cure after surgery and adjuvant chemotherapy was achieved in only 4.9%. For most patients, stability can be achieved with long-term chemotherapy, with or without surgery or interventional radiology, but the disorder remains chronic.

This report is the first collection of data on human alveolar echinococcosis in Europe. Our study confirms that an infection with this parasite is still dangerous. A low annual incidence persists in the previously known foci. However, case reports from regions remote from the core area of cases indicate that the disease is spreading. We therefore recommend that the occurrence of this potentially reemerging zoonosis should be continuously monitored in western and central Europe.
